# Diet-Induced Weight Loss Has No Effect on Psychological Stress in Overweight and Obese Adults: A Meta-Analysis of Randomized Controlled Trials

**DOI:** 10.3390/nu10050613

**Published:** 2018-05-14

**Authors:** Alison O. Booth, Xiaodan Wang, Anne I. Turner, Caryl A. Nowson, Susan J. Torres

**Affiliations:** Institute for Physical Activity and Nutrition, School of Exercise and Nutrition Sciences, Deakin University, Geelong, VIC 3220, Australia; alison.booth@deakin.edu.au (A.O.B.); swang17@gmail.com (X.W.); anne.turner@deakin.edu.au (A.I.T.); caryl.nowson@deakin.edu.au (C.A.N.)

**Keywords:** diet, weight loss, obesity, stress, adults, meta-analysis

## Abstract

The effect of weight loss on psychological stress is unknown. The study aimed to investigate the effect of diet-induced weight loss in overweight and obese adults on psychological measures of stress through a meta-analysis of randomized controlled trials (RCTs). Databases including Medline Complete, Embase and PsycINFO were searched up to February 2018 for diet-induced weight loss RCTs, which included self-reported assessment of psychological stress. The mean difference between the intervention and control group of changes in stress (intervention—baseline) was used. Ten RCTs were included with 615 participants (502 women, age range 20–80 years). Overall, there was no change in stress (mean difference −0.06, 95% CI: −0.17, 0.06, *p* = 0.33) and no change in the five studies with a significant reduction in weight in the intervention group compared to a control group that lost no weight (mean difference in weight −3.9 Kg, 95% CI: −5.51, −2.29, *p* < 0.0001; mean difference in stress 0.04, 95% CI: −0.17, 0.25, *p* = 0.71). For all analyses, there was low heterogeneity. The benefits of weight loss for those who are overweight and obese do not appear to either increase or reduce psychological stress at the end of the weight loss period.

## 1. Introduction

Obesity is a global epidemic and is increasing at an alarming rate [1], and is associated with comorbidities such as cardiovascular disease (CVD), type 2 diabetes and metabolic syndrome [2]. The causes of obesity are both diverse and complex, and can be attributed to physiological, environment, cultural, socioeconomic, psychological and genetic factors [3].

Stress can be defined as “the pattern of specific and nonspecific responses a person makes to stimulus events that disturb his or her equilibrium and tax or exceed his or her ability to cope” [4]. Psychological stress has been linked to the development of diseases such as CVD, cancer, depression and anxiety [5,6,7,8]. High levels of perceived psychological stress are also associated with factors related to unhealthy lifestyles such as a greater incidence of smoking, physical inactivity and consuming greater amounts of alcohol [9], as well as stimulating appetite for hedonic, highly palatable foods that are energy dense [10].

There is evidence to suggest that higher levels of stress may be associated with higher levels of body weight, potentially by increasing energy intake and decreasing physical activity, leading to a state of positive energy balance. A systematic review and meta-analysis of nine observational studies reported high rates of abdominal obesity (49%) in individuals with posttraumatic stress disorder [11]. A systematic review of prospective cohort studies found that there was moderate evidence to support the notion that relationship stress, perceived stress and distress was associated with the development of weight gain in adults [12]. However, a review of cross sectional studies examining the relationship between psychological workload, defined as high job demand and low influence [13], and body weight only reported a weak positive relationship [14].

Weight loss has been found to lower the risk for some comorbidities associated with obesity including CVD, type 2 diabetes, dyslipidaemia and hypertension [15]. A significant correlation between psychological stress and all domains of quality of life (QOL) was found in patients with hypertension [16]. Weight loss can also have beneficial effects on mental health including QOL, self-esteem and depression. Two studies have reported that weight loss improved QOL in obese individuals [17,18]. In a study by Fontaine et al. [19], weight loss of 8.6 kg over 13 weeks was associated with higher scores (enhanced QOL) for physical functioning, role-physical, general health, vitality and mental health domains of the SF-36. The authors proposed that these improvements were attributed to the increase in energy levels and participant’s ability to perform activities of daily living after weight loss. In a meta-analysis of 117 weight loss treatments assessing the effect on self-esteem and depression, only those that resulted in weight loss predicted improvements in self-esteem, whereas improvements in depression were independent of weight loss [20]. However, weight loss has also been reported to have detrimental effects on psychological well-being. Restricted energy diets for the treatment of overweight and obesity have been reported to increase fatigue and decrease vigour [21,22] and increase tension [22], which may be associated with feelings of deprivation and hunger. Considering the potential association between stress and body weight, it is important to understand the effect of weight loss on perceived level of psychological stress.

The aim of this systematic review and meta-analysis was to assess the effect of weight loss on psychological measures of stress in randomized controlled trials that induced weight loss by dietary restriction in overweight and obese adults and concurrently measured psychological stress.

## 2. Materials and Methods

The Preferred Reporting Items for Systematic Reviews and Meta-Analysis (PRISMA) guidelines were followed for this meta-analysis [23]. The protocol for this meta-analysis is available in PROSPERO (registration code: CRD42016039179).

### 2.1. Inclusion and Exclusion Criteria

The meta-analysis was limited to peer reviewed randomized controlled trials (RCTs) that included at least one diet-induced weight loss intervention, that targeted overweight and/or obese adult men and/or women (≥18 years, Body Mass Index (BMI) ≥ 25 kg/m^2^), reported significant weight loss in at least one arm and baseline, end and/or change in stress levels. We included studies that measured body weight and/or BMI as the primary outcome indicator of dietary restriction. Studies were required to report a psychological measure of self-reported stress using one of the established measures as defined by Figueroa-Fankhanel [24].

We excluded studies with pregnant and/or lactating women. Studies utilising a combination of diet and other means of weight loss were excluded: psychotherapies (e.g., Cognitive Behaviour Therapy) as these may have independent effects on psychological stress; drug therapy to induce weight loss; and surgery (e.g., gastric banding, gastric bypass, or gastroplasty) as this population group has a high prevalence of psychopathology and personality disturbances [25]. Studies with structured and monitored exercise regimens were excluded from the meta-analysis as exercise is known to improve stress [26], but those that encouraged participants to undertake physical activity to maintain a healthy lifestyle were included.

### 2.2. Search Strategy

Online literature database searches were performed in Medline Complete, AMED, CINAHL complete, PsycARTICLES, PsycBOOKS, PsycEXTRA, PsycINFO, Psychology and Behavioral Sciences Collection, Global Health, Embase, Scopus, EMB reviews, APAIS-Health, Health & Society, PsychiatrOnline.org, Health collection, and trove.nla.gov.au (grey literature). Searches were restricted to human studies that were published in English language, but not limited by publication date up to February 2018. The following terms were used: weight loss, weight management, weight reduction, overweight, obes*, obesity treat*, dietary intervention, diet*, diet reduc*, stress, psyc* distress, mental health, psyc* and psyc* health. From the combination of the above search terms and the Medical Subject Headings (MeSH) database filters, relevant journal articles were retrieved.

### 2.3. Search Results and Selection of Studies

All search results generated by the database searches were exported into a reference management system (EndNote X8) and duplicates were removed. Two investigators independently screened the search results against study inclusion and exclusion criteria. Titles and abstracts were first reviewed to determine the eligibility for full text assessment. The full texts of potentially qualifying studies were then retrieved and reviewed. Disagreements between the independent investigators were resolved by discussion and consensus.

### 2.4. Risk for Bias

The risk for bias was assessed using the methods outlined in the Cochrane Handbook for Reviews of Interventions [27]. The following were assessed: random sequence generation; allocation concealment; blinding of participants, personnel and outcome assessment; incomplete outcome data and selective reporting.

### 2.5. Data Extraction

Eligible studies were reviewed, and the following data were extracted by two investigators: first author’s name and year of publication; number of participants in the intervention and control groups, age, baseline BMI; dietary intervention and control, method used to measure psychological stress, study duration; change in weight and stress. Where one study reported data from the Profile of Mood States (POMS) and Spielberger State Trait Anxiety Inventory (STAI) [28], only the (STAI) results were included in the analysis to allow for greater consistency between included studies. Where mean changes were reported graphically with variance [28], the graph was magnified 600× and values were estimated by the author (AOB). Where variance was reported as SE, SD was calculated. For studies that reported stress at end, change from baseline standard deviation was imputed using a correlation coefficient [27,29,30,31,32]. A correlation value of r = 0.6 was used as it is considered a conservative estimate from reliability studies while taking into consideration longer time periods between baseline and final measurements of the included studies [27].

### 2.6. Statistical Analysis

Review Manager (RevMan version 5.3) was used to conduct the meta-analysis. Initial analysis compared stress at end with stress at baseline for each trial arm of the studies where all groups lost weight. A second analysis included all dietary interventions comparing stress at end with stress at baseline for all trial arms to determine if participating in a study affected stress outcome. Sub-analyses were performed for studies reporting a difference in weight change between groups and for all trials that compared intervention groups that lost weight with control groups that did not lose weight using change in stress as the outcome. Random effects models with standardized mean differences, as recommended by the Cochrane Collaboration [27], were used for all analyses. All treatment effects are presented with 95% confidence intervals (CI). Heterogeneity was assessed using Q-statistics (Chi^2^) and *I*^2^ [33]. Funnel plots were used to detect the possibility of publication bias, and Egger’s regression test [34] to measure funnel plot asymmetry was performed using Stata Statistical Software (release 15; College Station, TX: StataCorp LLC).

## 3. Results

**Figure 1:**
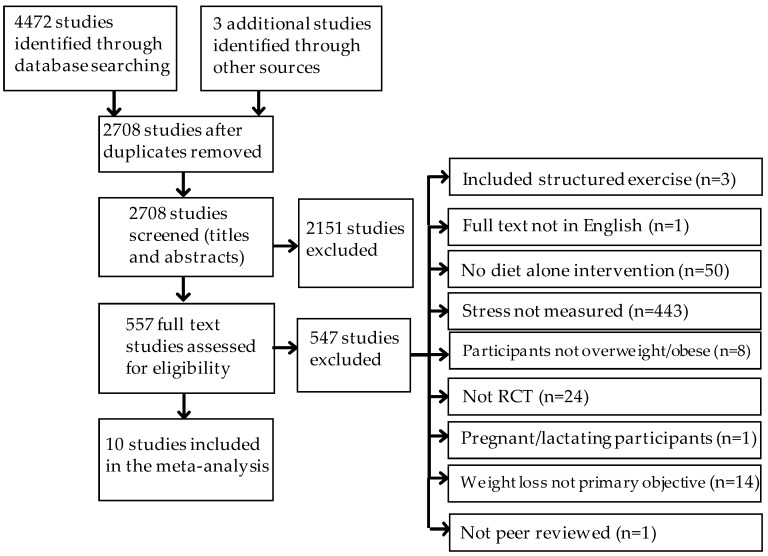
Study selection flow chart and reasons for full-text screening study exclusion.

Figure 1 shows the process by which the included studies were identified. We identified 4472 potential RCTs from the electronic search and a further three studies through manual searches of relevant articles. Removing duplicates left 2708 studies, of which 2151 were assessed to not meet the inclusion criteria. Abstracts and full text articles for the remaining 557 studies were judged as requiring full review. Of these remaining studies, 10 trials met the inclusion criteria and were included in the meta-analysis.

### 3.1. General Characteristics of the Selected Studies

The sample size, age, baseline BMI, dietary intervention, stress measure, study duration, weight loss and effect on stress in the intervention and control groups in each of the 10 RCTs are described in Table 1. Sample sizes ranged from 16 to 205 with a total of 615 participants, age range 20–80 years. The duration of the trials ranged from 3 weeks to 12 months. The most common measure of psychological stress was the STAI [35], which was used in 7 trials [4 reported STAI-S (State) and 3 reported STAI-ST (State and Trait combined)], followed by the Perceived Stress Scale [36], which was used in 2 trials, one study used the POMS [37] and one study used the Positive and Negative Affect Schedule (PANAS) [38]. The STAI is a respondent-based questionnaire with a cognitive and emotional theoretical underpinning that measures State anxiety, how respondents feel right now, and Trait anxiety, how respondents generally feel [24,39]. Scores range from 20 to 80 with higher scores indicating higher levels of anxiety [39]. The Perceived Stress Scale is respondent based and has a cognitive, emotional and environmental theoretical underpinning [24]. The original 14-item scale measures the degree to which situations in one’s life are appraised as stressful [36]. Scores range from 0 to 4 with larger scores indicating greater perceived stress. The POMS is a respondent-based questionnaire with an emotional theoretical underpinning [24]. The 37 item POMS is a validated tool designed to assess current mood and changes in mood state. Participants rate their mood state on a five-point Likert scale (0 = not at all, 4 = extremely) which best described how they had been feeling during the past week, with lower scores indicating better mood [40]. The PANAS is respondent based and has an emotional and environmental underpinning [24]. The instrument comprises two 10-item scales, providing independent measurements of positive and negative affect [38]. Five trials reported stress as a primary outcome [28,30,31,32,41] and five trials reported stress as a secondary outcome [29,32,42,43,44,45]. For the study that reported both POMS and STAI results [28], only the STAI results were included in the analysis. In the studies that separated STAI-S and STAI-T results, the STAI-S was selected for inclusion [29,43].

### 3.2. Risk for Bias Assessment Summary

The risk for bias assessments are described in Table 2. Overall, across the ten intervention studies, six had low risk and four had high risk for bias.

### 3.3. Effect of Weight Loss on Stress

**Figure 2:**
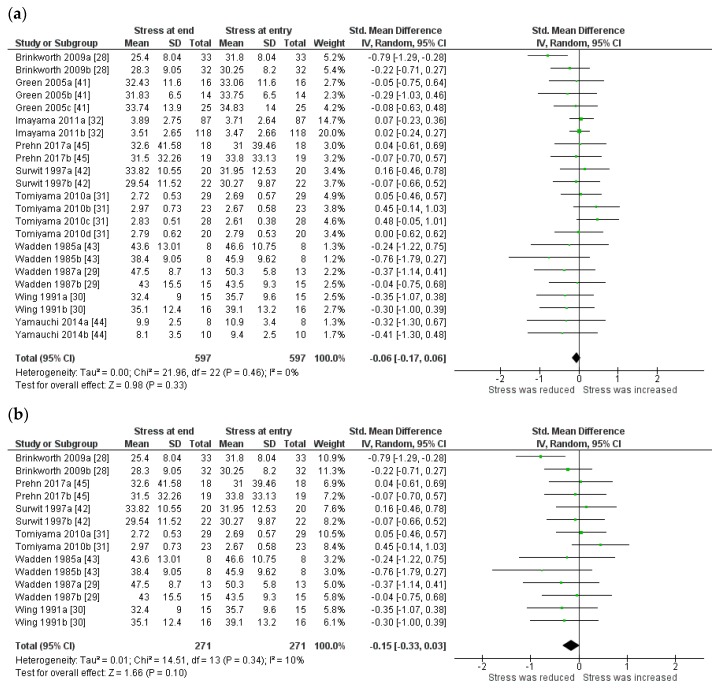
Forest plot displaying standard mean difference and 95% confidence intervals (CI) for the effect of weight loss on stress (**a**) in all trial arms from studies and (**b**) in all trial arms from studies that resulted in dietary-induced weight loss. ^a,b,c,d^ Multiple trial arms.

Meta-analysis was performed in all ten studies representing 22 trial arms including those that lost weight and maintained weight (Figure 2a). Overall, there was no significant change in stress (mean difference −0.06, 95% CI = −0.17, 0.06, *p* = 0.33) (Q = 22.0, *df* = 22 (*p* = 0.46) *I*^2^ = 0% (Figure 2a). In a subgroup analysis of six studies where there was a significant reduction in weight in all trial arms, no improvement in stress was reported (mean difference −0.15 (SD), 95% CI = −0.33, 0.03, *p* = 0.10, (Q = 14.5, *df* = 13 (*p* = 0.34) *I*^2^ = 10% (Figure 2b).

**Figure 3:**
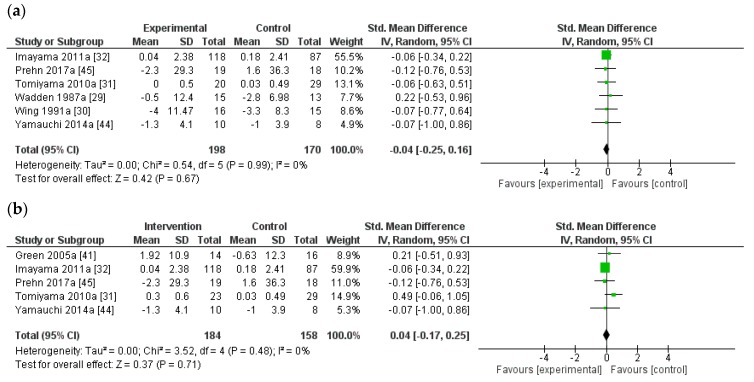
Forest plot displaying standard mean difference and 95% confidence intervals (CI) for measures of psychological stress: trials with (**a**) significant difference in weight change between groups (mean difference −5.4 ± 17.1 kg) and (**b**) significant weight loss in intervention group compared to no weight loss in control group (mean difference −3.9 ± 15.2 kg).

Meta-analysis of the effect of weight loss on stress was performed in six studies where there was a significant difference in weight reduction between groups (mean difference −5.4 ± 17.1 kg). No improvement in stress was reported (mean difference −0.04 (SD), 95% CI = −0.25, 0.16 *p* = 0.67) (Q = 0.5, *df* = 5 (*p* = 0.99) *I*^2^ = 0% (Figure 3a). When the analysis was limited to five studies with statistically significant weight loss compared to a control group that lost no weight (mean difference −3.9 ± 15.2 kg), there were still no improvements seen in stress (mean difference 0.04, 95% CI = −0.17, 0.25, *p =* 0.71) (Q = 3.5, *df =* 5 (*p* = 0.48) *I*^2^ = 0% (Figure 3b). For all analyses, we observed low heterogeneity (Figure 2 and Figure 3).

### 3.4. Test for Publication Bias

There was no statistical evidence for publication bias across all analyses (for all trial arms Eggers regression, *p* = 0.123; all RCTs that resulted in weight loss in all trial arms: Eggers regression, *p* = 0.860; all RCTs that resulted in significant between group weight change Eggers regression, *p* = 0.655 and; all RCTs where the intervention group resulted in weight loss compared to no weight change in the control group Eggers regression *p* = 0.612 (Supplementary Figure S1a–d).

## 4. Discussion

To our knowledge, this is the first meta-analysis to analyse results from RCTs to assess the effect of diet-induced weight loss on stress in overweight and obese adults. There were only five studies that reported significantly greater weight loss in the intervention group compared to a control group that lost no weight (range of 0.9–12.3 kg reduction with one study reporting a reduction of 8.5%) enabling a more robust analysis of the effect of weight loss on stress [31,32,41,44,45]. Overall, the results from this meta-analysis revealed that there were no effects on stress, either positive or negative of undertaking a dietary weight loss program within a trial setting. Furthermore, when we examined seven studies representing 14 trial arms that all reported significant weight loss, there was no overall effect on levels of psychological stress. These findings are in agreement with our recent review, where we found no strong evidence to indicate that diet-induced weight loss had a detrimental effect on anxiety [46].

Previous research has shown that energy restriction over long periods to induce weight loss may impose feelings of stress or affect QOL [28,47]. Many studies included in this meta-analysis required participants to consume very low-calorie diets which has been shown to decrease vigour and increase tension [21,22,31]. In the trial by Tomiyama et al. [31], those who monitored and restricted (received instructions on how to complete a daily food diary so they monitored their energy intake) or *monitored* all their dietary intake (provided with all the food that they consumed over the course of the study) reported an increase in perceived stress, whereas those participants who only restricted their dietary intake reported no change in perceived stress [31]. The authors have suggested that the continual monitoring of diet may have been irritating for the participants. However, despite the potential negative effect that diet induced weight loss might have on an individual’s level of stress, overall the current meta-analysis found that weight loss did not have a detrimental effect on the level of psychological stress.

Conversely, we should also consider the possibility that involvement in a dietary intervention might be having a positive impact on stress rather than the weight loss per se due to the additional contact and support participants receive from research staff. In the study by Imayama et al. [32], participants in the 12-month reduced calorie dietary weight loss group initially had individual sessions with the dietitian and then met weekly with small groups. The study reported by Brinkworth et al. also required participants to follow a 12-month weight loss study, which involved regular contact with a dietitian [28]. It is possible that motivating research staff may have had a positive influence on the participants’ moods and feelings of self-worth and self-confidence. It has been reported in weight loss studies that mood improves after only a short period [48], which may be the result of additional social support provided by researchers and participants taking control of their lifestyle and dietary habits. Nevertheless, in the current meta-analysis, we observed no overall change in self-reported stress levels measured by validated instruments.

In this meta-analysis, we included studies with weight loss that ranged from −0.9 kg [31] to −20.5 kg [29]. The impact of weight loss might have a greater effect on stress in those with a higher baseline BMI. There was a greater improvement in stress in the Brinkworth et al. study [28] which recruited obese individuals compared to the Green et al. study [41] which recruited overweight individuals. However, in the study by Wadden et al. [29], there was no effect on stress even with a large average weight loss of 20.5 kg and baseline BMI in the obese range. Due to lack of available data (difference in weight change between the groups), we were unable to conduct a meta-regression to determine if there were an association between the change in body weight and change in stress. However, the findings from this meta-analysis would appear to indicate that across a broad range of weight loss from 0.9 to 20.5 kg, there is no impact on psychological stress.

It is also important to consider the duration of studies when interpreting the findings from this meta-analysis. It is possible that there may be an optimal study duration which would result in an improvement in stress. However, when we examined the five studies that reported significantly greater weight loss in the intervention group compared to a control group that lost no weight, we found that there was no effect of weight loss on stress with study durations ranging from 3 weeks to 12 months [31,32,41,44,45].

In this meta-analysis, we included studies that assessed stress using questionnaires that were respondent-based, and with different theoretical underpinnings, for example emotional or cognitive, as defined by Figueroa-Fankhanel (2016) [24]. This search only yielded ten studies that were eligible to be included in this meta-analysis, with inclusion of three different measures of psychological stress. Due to the varying measures and scales used to assess stress [24], standardized mean differences were used in the analysis.

An important strength of this meta-analysis is that it only included RCTs. Furthermore, low heterogeneity was noted indicating that the variation in the true effect size across studies is low. However, only five of the ten studies were designed to specifically assess the effect of weight loss on stress as the primary outcome [28,30,31,32,41]. There were a number of methodological flaws inherent in the studies that need to be highlighted. In a number of studies examined, stress was measured at baseline and at the end of the study (for example 6 months or 12 months) [28,29,30,32], which does not give an indication of the patterns of change in stress throughout the intervention. There may have been improvements in stress in the early part of the intervention that were not detected in some studies [29].

## 5. Conclusions

This systematic review and meta-analysis of randomized controlled clinical trials showed that weight loss induced by dietary restriction in adult overweight and obese men and women does not have a beneficial or detrimental effect on self-reported psychological stress. However, further randomized controlled trials with robust methods measuring stress, utilising solely a dietary approach with sufficient sample sizes and study durations which result in significant weight loss would be required before we can draw any final conclusions about the effect of weight loss on stress. It is recommended that stress is measured at a number of time points throughout an intervention, to give a more accurate representation of the pattern of changes over time.

## Figures and Tables

**Table 1:** Eligible randomized controlled trial studies investigating the effect of diet induced weight loss on psychological stress in overweight and obese adults.

	Participants			Study Design			Outcomes	
Study	*N*	Age (mean ± SD) or range (years)	Baseline BMI (kg/m^2^)	Intervention	Stress measure	Duration	Weight change	Stress change
Brinkworth et al. 2009 [28]	65 men and women with abdominal obesity #	50.0 ± 8.2	33.7 ± 4.1	IG: Isocaloric conventional high carbohydrate low-fat diet CG: Energy restricted very-low carbohydrate, high-fat diet	POMS STAI-ST	12 months	IG: −13.7 kg * CG: −13.7 kg *	IG: improvement in STAI-ST vs CG *p* < 0.05 between groups
Green et al. 2005 [41]	55 overweight women	20–45	IG: 29.3 ± 6.5 CG1: 28.2 ± 4.1 CG2: 26.9 ± 6.5	IG: Hypocaloric supported diet CG1: Hypocaloric unsupported diet CG2: Usual diet	STAI-S	8 weeks	IG: −2.7 kg * CG1: −2.2 kg * CG2: −0.05 kg *	IG: no change CG1: no change
Imayama et al. 2011 [32]	205 overweight and obese women	IG: 58.1 ± 5.9 CG: 57.4 ± 4.4	IG: 31.0 ± 3.9 CG: 30.7 ± 3.9	IG: Reduced calorie dietary weight loss (1200–2000 kcal/day) CG: Usual diet	Perceived stress scale	12 months	IG: −8.5% compared with CG *p* < 0.01 between groups	IG: no change CG: increased *
Prehn et al. 2017 [45]	37 obese women	IG: 61 ± 4 CG: 61 ± 6	IG: 35.0 (3.7) CG: 34.7 (4.3)	IG: Calorie restriction CG: Usual diet	PANAS STAI-ST	12 weeks	IG: −12.3 kg CG: −0.2 kg CG: not reported	Positive PANAS IG: no change CG: decreased * Negative PANAS IG: no change CG: no change STAI-ST IG: no change CG: no change
Surwit et al. 1997 [42]	42 women 130–200% of ideal body weight	IG: 40.6 ± 8.2 CG: 40.3 ± 7.3	IG: 35.9 ± 4.8 CG: 34.9 ± 4.4	IG: Low-fat, high-sucrose hypoenergetic diet CG: Low-fat, low-sucrose hypoenergetic diet	STAI-S	6 weeks	IG: −6.9 kg * CG: −7.4 kg *	IG: no change CG: no change
Tomiyama et al. 2010 [31]	99 women not underweight	NA	IG: 25.8 ± 3.6 CG1: 24.4 ± 4.0 CG2: 24.9 ± 4.5 CG3: 24.1 ± 3.4	IG: Monitoring + restricting (1200 kcal/day) CG1: Monitoring only CG2: Restricting only (1200 kcal/day) CG3: Control	Perceived stress scale	3 weeks	IG:-0.9 kg CG1:-1.2 kg CG2:-0.9 kg CG3:+2.2 kg *p* < 0.05 between groups	IG: increased * CG1:increased * CG2: no change CG3: no change
Wadden et al. 1985 [43]	16 moderately overweight men and women #	38.1	NA	IG: Protein-sparing modified fast (450 kcal/day) CG: Protein-formula liquid diet (420 kcal/day)	STAI-ST	4 weeks	IG: −8.7 kg * CG: −7.3 kg *	IG: decreased * CG: decreased
Wadden et al. 1987 [29]	Obese men (5) and women (30)	44.1 ± 8.7 (women) 42.3 ± 11.6 (men)	NA	IG: 500 kcal protein-sparing modified fast CG: 1200 kcal balanced diet	STAI-ST	25 weeks	IG: −20.5 kg * CG: −15.7 kg * *p* < 0.06 between groups	IG: no change CG: no change
Wing et al. 1991 [30]	18 men and 25 women >30% above ideal body weight	35–70	NA	IG: VLCD: 1 to 4 weeks—1000–1500 cal/day, 5 to 12 weeks—400 cal/day, 13 to 20 weeks—1000–1500 cal/day CG: Balanced diet: 1000–1500 cal/day	STAI-ST	20 weeks	IG: −18.6 kg * CG: −10.1 kg * *p* < 0.003 between groups	IG: decreased * CG: decreased *
Yamauchi et al. 2014 [44]	Overweight and obese men (9) and women (9)	IG: 55.8 ± 10.4 CG: 59.0 ± 11.9	IG: 27.6 ± 3.8 CG: 28.4 ± 2.4	IG: Lifestyle modification including healthy plate CG: Usual diet	POMS	3 months	IG: −3.7 kg * CG: −0.1 kg *p* < 0.001 between groups	IG: no change CG: no change

^#^ Number of men and women not reported; IG: Intervention Group; CG: Control Group; STAI-ST: Spielberger State-Trait Anxiety Inventory; NA: Not Available; PANAS: Positive and Negative Affect Schedule; VLCD: Very Low-Calorie Diet; POMS: Profile of Mood States. * *p* < 0.05 within group.

**Table 2:** Assessment of Risk of Bias in the included studies using Cochrane Criteria [27].

Study	Random Sequence Generation	Allocation Concealment	Blinding of Participants and Personnel	Blinding of Outcome Assessment	Incomplete Outcome Data	Selective Reporting	Other Bias	Overall Risk of Bias
Brinkworth et al. 2009 [28]	Low risk	High risk	Low risk	Low risk	High risk	High risk	Low risk	High risk
Green et al. 2005 [41]	Low risk	High risk	Low risk	Low risk	High risk	High risk	Low risk	High risk
Imayama et al. 2011 [32]	High risk	High risk	Low risk	Low risk	Low risk	Low risk	Low risk	Low risk
Prehn et al. 2017 [45]	High risk	Low risk	Low risk	Low risk	Unclear	Low risk	Low risk	Low risk
Surwit et al. 1997 [42]	High risk	High risk	Low risk	Low risk	High risk	High risk	Low risk	High risk
Tomiyama et al. 2010 [31]	Low risk	High risk	Low risk	Low risk	Low risk	Low risk	Low risk	Low risk
Wadden et al. 1985 [43]	High risk	High risk	Low risk	Low risk	High risk	High risk	Low risk	High risk
Wadden et al. 1987 [29]	Low risk	High risk	Low risk	Low risk	Low risk	Low risk	Low risk	Low risk
Wing et al. 1991 [30]	Low risk	High risk	Low risk	Low risk	Low risk	Low risk	Low risk	Low risk
Yamauchi et al. 2014 [44]	Low risk	High risk	Low risk	Low risk	Low risk	Low risk	Low risk	Low risk

## Supplementary Materials

The following are available online at http://www.mdpi.com/2072-6643/10/5/613/s1, Figure S1a–d, Funnel plots displaying standard mean difference and standard error (SE) for the effect of weight loss on stress.

## References

[1] Swinburn B.A., Sacks G., Hall K.D., McPherson K., Finegood D.T., Moodie M.L., Gortmaker S.L. (2011). The global obesity pandemic: Shaped by global drivers and local environments. Lancet.

[2] World Health Organisation (2011). Global Strategy on Diet, Physical Activity and Health.

[3] World Health Organisation (2000). Obesity: Preventing and Managing the Global Epidemic.

[4] American Psychiatric Association (2013). Diagnostic and Statistical Manual of Mental Disorders (DSM-5).

[5] Krantz D.S., McCeney M.K. (2002). Effects of psychological and social factors on organic disease: A critical assessment of research on coronary heart disease. Annu. Rev. Clin. Psychol..

[6] Kruk J., Aboul-Enein H.Y. (2004). Psychological stress and the risk of breast cancer: A case–control study. Cancer Detect. Prev..

[7] Hammen C. (2005). Stress and Depression. Annu. Rev. Clin. Psychol..

[8] Spielberger C.D., Sarason I.G., Strelau J., Brebner J.M. (2014). Stress and Anxiety.

[9] Rod N.H., Gronbaek M., Schnohr P., Prescott E., Kristensen T.S. (2009). Perceived stress as a risk factor for changes in health behaviour and cardiac risk profile: A longitudinal study. J. Intern. Med..

[10] Torres S.J., Nowson C.A. (2007). Relationship between stress, eating behavior, and obesity. Nutrition.

[11] Rosenbaum S., Stubbs B., Ward P.B., Steel Z., Lederman O., Vancampfort D. (2015). The prevalence and risk of metabolic syndrome and its components among people with posttraumatic stress disorder: A systematic review and meta-analysis. Metabolism.

[12] Bergmann N., Gyntelberg F., Faber J. (2014). The appraisal of chronic stress and the development of the metabolic syndrome: A systematic review of prospective cohort studies. Endocr. Connect..

[13] Overgaard D., Gamborg M., Gyntelberg F., Heitmann B.L. (2004). Psychological workload is associated with weight gain between 1993 and 1999: Analyses based on the Danish Nurse Cohort Study. Int. J. Obes..

[14] Overgaard D., Gyntelberg F., Heitmann B.L. (2004). Psychological workload and body weight: Is there an association? A review of the literature. Occup. Med..

[15] Rippe J.M., Crossley S., Ringer R. (1998). Obesity as a chronic disease: Modern medical and lifestyle management. J. Am. Diet. Assoc..

[16] Baune B., Aljeesh Y. (2006). The association of psychological stress and health related quality of life among patients with stroke and hypertension in Gaza Strip. Ann. Gen. Psychiatry.

[17] Pan H.J., Cole B.M., Geliebter A. (2011). The benefits of body weight loss on health-related quality of life. J. Chin. Med. Assoc..

[18] Kaukua J., Pekkarinen T., Sane T., Mustajoki P. (2003). Health-related quality of life in obese outpatients losing weight with very-low-energy diet and behaviour modification: A 2-y follow-up study. Int. J. Obes. Relat. Metab. Disord..

[19] Fontaine K.R., Barofsky I., Andersen R.E., Bartlett S.J., Wiersema L., Cheskin L.J., Franckowiak S.C. (1999). Impact of weight loss on health-related quality of life. Qual. Life Res..

[20] Blaine B.E., Rodman J., Newman J.M. (2007). Weight loss treatment and psychological well-being: A review and meta-analysis. J. Health Psychol..

[21] Buffenstein R., Karklin A., Driver H.S. (2000). Beneficial physiological and performance responses to a month of restricted energy intake in healthy overweight women. Physiol. Behav..

[22] Degoutte F., Jouanel P., Begue R.J., Colombier M., Lac G., Pequignot J.M., Filaire E. (2006). Food restriction, performance, biochemical, psychological, and endocrine changes in judo athletes. Int. J. Sports Med..

[23] Moher D., Liberati A., Tetzlaff J., Altman D.G., Group P. (2009). Preferred reporting items for systematic reviews and meta-analyses: The PRISMA statement. BMJ.

[24] Figueroa-Fankhanel F. (2014). Measurement of stress. Psychiatr. Clin. N. Am..

[25] Glinski J., Wetzler S., Goodman E. (2001). The psychology of gastric bypass surgery. Obes. Surg..

[26] Wycherley T.P., Clifton P.M., Noakes M., Brinkworth G.D. (2014). Weight loss on a structured hypocaloric diet with or without exercise improves emotional distress and quality of life in overweight and obese patients with type 2 diabetes. J. Diabetes Investig..

[27] Higgins J.P.T., Green S. (2011). Cochrane Handbook for Systematic Reviews of Interventions.

[28] Brinkworth G.D., Buckley J.D., Noakes M., Clifton P.M., Wilson C.J. (2009). Long-term effects of a very low-carbohydrate diet and a low-fat diet on mood and cognitive function. Arch. Intern. Med..

[29] Wadden T.A., Stunkard A.J., Day S.C., Gould R.A., Rubin C.J. (1987). Less food, less hunger: Reports of appetite and symptoms in a controlled study of a protein-sparing modified fast. Int. J. Obes..

[30] Wing R.R., Marcus M.D., Blair E.H., Burton L.R. (1991). Psychological responses of obese type II diabetic subjects to very-low-calorie diet. Diabetes Care.

[31] Tomiyama A.J., Mann T., Vinas D., Hunger J.M., Dejager J., Taylor S.E. (2010). Low calorie dieting increases cortisol. Psychosom. Med..

[32] Imayama I., Alfano C.M., Kong A., Foster-Schubert K.E., Bain C.E., Xiao L., Duggan C., Wang C.Y., Campbell K.L., Blackburn G.L. (2011). Dietary weight loss and exercise interventions effects on quality of life in overweight/obese postmenopausal women: A randomized controlled trial. Int. J. Behav. Nutr. Phys. Act..

[33] Borenstein M., Higgins J.P., Hedges L.V., Rothstein H.R. (2017). Basics of meta-analysis: I2 is not an absolute measure of heterogeneity. Res. Synth. Methods.

[34] Rothsein H., Sutton A., Borenstein M. (2005). Publication Bias in Meta-Analysis: Prevention, Assessment and Adjustments.

[35] Spielberger G., Gorush R., Lusshene R. (1970). The State-Trait Anxiety Inventory.

[36] Cohen S., Kamarck T., Mermelstein R. (1983). A global measure of perceived stress. J. Health Soc. Behav..

[37] Shacham S. (1983). A shortened version of the Profile of Mood States. J. Personal. Assess..

[38] Watson D., Clark L.A., Tellegen A. (1988). Development and validation of brief measures of positive and negative affect: The PANAS scales. J. Pers. Soc. Psychol..

[39] Spielberger C.D., Gorsuch R.L., Lushene R., Vagg P.R., Jacobs G.A. (1983). Manual for the State-Trait Anxiety Inventory.

[40] Baker F., Denniston M., Zabora J., Polland A., Dudley W.N. (2002). A POMS short form for cancer patients: Psychometric and structural evaluation. Psychooncology.

[41] Green M.W., Elliman N.A., Kretsch M.J. (2005). Weight loss strategies, stress, and cognitive function: Supervised versus unsupervised dieting. Psychoneuroendocrinology.

[42] Surwit R.S., Feinglos M.N., McCaskill C.C., Clay S.L., Babyak M.A., Brownlow B.S., Plaisted C.S., Lin P.H. (1997). Metabolic and behavioral effects of a high-sucrose diet during weight loss. Am. J. Clin. Nutr..

[43] Wadden T.A., Stunkard A.J., Brownell K.D., Day S.C. (1985). A comparison of two very-low-calorie diets: Protein-sparing-modified fast versus protein-formula-liquid diet. Am. J. Clin. Nutr..

[44] Yamauchi K., Katayama T., Yamauchi T., Kotani K., Tsuzaki K., Takahashi K., Sakane N. (2014). Efficacy of a 3-month lifestyle intervention program using a Japanese-style healthy plate on body weight in overweight and obese diabetic Japanese subjects: A randomized controlled trial. Nutr. J..

[45] Prehn K., Jumpertz von Schwartzenberg R., Mai K., Zeitz U., Witte A.V., Hampel D., Szela A.M., Fabian S., Grittner U., Spranger J. (2017). Caloric Restriction in Older Adults-Differential Effects of Weight Loss and Reduced Weight on Brain Structure and Function. Cereb. Cortex.

[46] Eyres S.L., Turner A.I., Nowson C.A., Torres S.J. (2014). Does diet-induced weight change effect anxiety in overweight and obese adults?. Nutrition.

[47] Laederach-Hofmann K., Kupferschmid S., Mussgay L. (2002). Links between body mass index, total body fat, cholesterol, high-density lipoprotein, and insulin sensitivity in patients with obesity related to depression, anger, and anxiety. Int. J. Eat. Disord..

[48] Wadden T.A., Steen S.N., Wingate B.J., Foster G.D. (1996). Psychosocial consequences of weight reduction: How much weight loss is enough?. Am. J. Clin. Nutr..

