# Supplementary material for: Diet-Induced Weight Loss Has No Effect on Psychological Stress in Overweight and Obese Adults: A Meta-Analysis of Randomized Controlled Trials
Source: Nutrients. 2018 May 14;10(5):613. doi: 10.3390/nu10050613 (PMC5986493; doi:10.3390/nu10050613)
Supplement: Supplementary file 1 [file nutrients-10-00613-s001.docx]

(a)


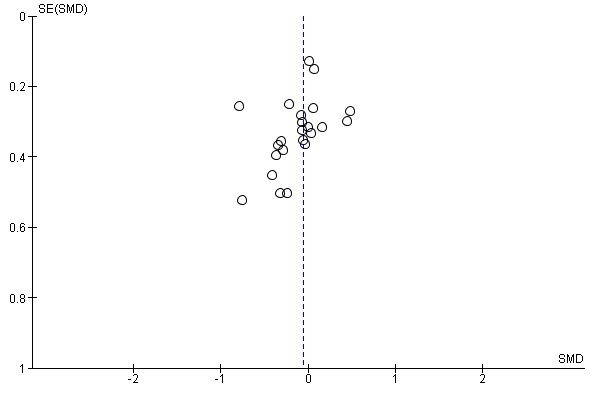


(b)


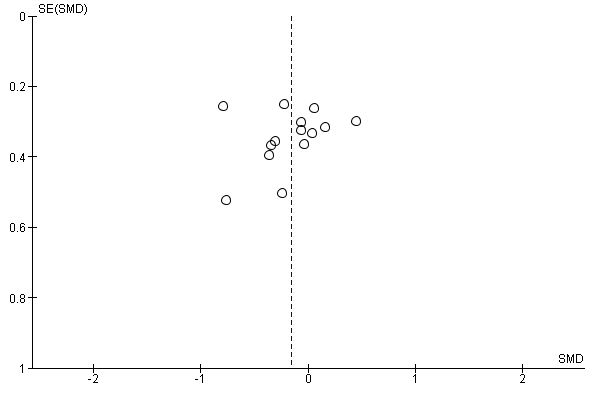


(c)


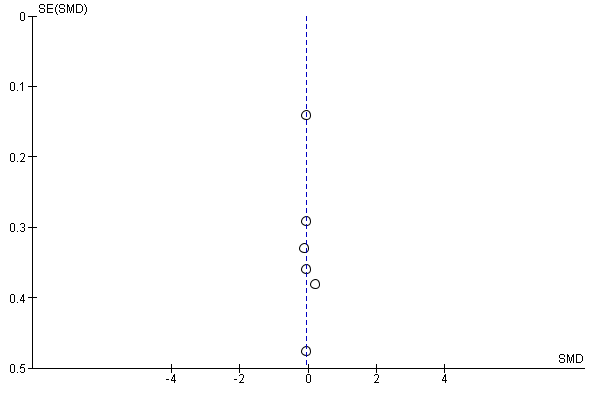


(d)


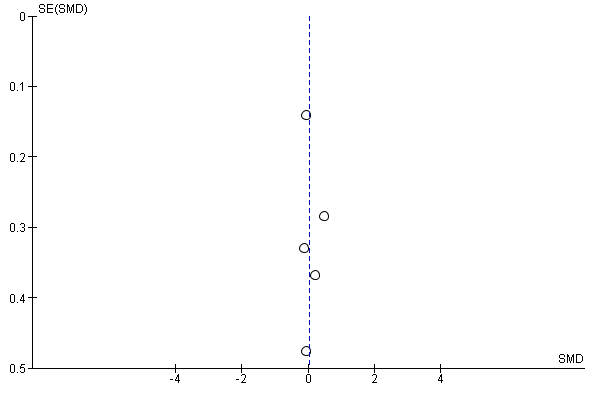


Supplementary Figure 1 Funnel plots displaying standard mean difference and standard error (SE) for the effect of weight loss on stress. Mid vertical line represents the zero mean difference or zero effect size. (a) Funnel plot of all 10 trials, Egger’s regression, *p* = 0.123 indicates no publication bias. (b) Funnel plot of all RCTs that resulted in weight loss in all trial arms, Egger’s regression, *p* = 0.860 indicates no publication bias. (c) Funnel plot of studies that resulted in a significant difference in weight change between groups, Egger’s regression, *p* = 0.655 indicates no publication bias. (d) Funnel plot of studies where the intervention group resulted in weight loss compared to no weight change in the control group, Egger’s regression, *p* = 0.612 indicates no publication bias.
